# Dietary Fatty Acid Composition Alters Gut Microbiome in Mice with Obesity-Induced Peripheral Neuropathy

**DOI:** 10.3390/nu17040737

**Published:** 2025-02-19

**Authors:** Mohamed H. Noureldein, Amy E. Rumora, Samuel J. Teener, Diana M. Rigan, John M. Hayes, Faye E. Mendelson, Andrew D. Carter, Whitney G. Rubin, Masha G. Savelieff, Eva L. Feldman

**Affiliations:** 1 Department of Neurology, University of Michigan, Ann Arbor, MI 48109, USA; 2 NeuroNetwork for Emerging Therapies, University of Michigan, Ann Arbor, MI 48109, USA; 3 Department of Neurology, Columbia University, New York, NY 10032, USA; 4 Department of Biomedical Sciences, University of North Dakota, Grand Forks, ND 58202, USA

**Keywords:** fecal microbiota transplantation, inflammation, monounsaturated fatty acid, prediabetes, saturated fatty acid

## Abstract

Background: Peripheral neuropathy (PN), a complication of diabetes and obesity, progresses through a complex pathophysiology. Lifestyle interventions to manage systemic metabolism are recommended to prevent or slow PN, given the multifactorial risks of diabetes and obesity. A high-fat diet rich in saturated fatty acids (SFAs) induces PN, which a diet rich in monounsaturated fatty acids (MUFAs) rescues, independent of weight loss, suggesting factors beyond systemic metabolism impact nerve health. Interest has grown in gut microbiome mechanisms in PN, which is characterized by a distinct microbiota signature that correlates with sciatic nerve lipidome. Methods: Herein, we postulated that SFA- versus MUFA-rich diet would impact gut microbiome composition and correlate with PN development. To assess causality, we performed fecal microbiota transplantation (FMT) from donor mice fed SFA- versus MUFA-rich diet to lean recipient mice and assessed metabolic and PN phenotypes. Results: We found that the SFA-rich diet altered the microbiome community structure, which the MUFA-rich diet partially reversed. PN metrics correlated with several microbial families, some containing genera with feasible mechanisms of action for microbiome-mediated effects on PN. SFA and MUFA FMT did not impact metabolic phenotypes in recipient mice although SFA FMT marginally induced motor PN. Conclusions: The involvement of diet-mediated changes in the microbiome on PN and gut–nerve axis may warrant further study.

## 1. Introduction

Peripheral neuropathy (PN) is damage to the peripheral nerves that arises as a diabetic complication, even in type 2 diabetes patients with good glycemic control [1], suggesting risk factors beyond hyperglycemia. Indeed, obesity and other components of the metabolic syndrome, a constellation of metabolic dysfunctions, are also PN risk factors, even independent of glycemic control [1]. Several mechanisms contribute to PN pathophysiology, including impaired nerve metabolism, bioenergetics failure, inflammation, and oxidative stress [2]; however, disease pathways remain incompletely understood and PN currently lacks disease-modifying, mechanism-based therapies.

Lifestyle interventions to manage weight and systemic metabolic profiles have emerged as a viable route to prevent or slow PN, given multifactorial risks from obesity and dyslipidemia as well as diabetes. In preclinical models of obesity, we have shown that placing mice on a high-fat diet (HFD) rich in saturated fatty acids (SFAs) induces obesity and PN along with changes in nerve lipidome [3]. Conversely, dietary reversal by switching mice back to a low-fat standard diet (SD) promotes weight loss and reverses PN. Moreover, mimicking a Mediterranean diet by replacing fat calories from SFAs with monounsaturated fatty acids (MUFAs) also rescues PN in mice [4], without affecting weight, suggesting that factors beyond systemic metabolism may impact nerve health.

Gut microbiome-mediated mechanisms in PN pathophysiology have gained traction recently [5]. Obesity [6] and SFA intake [7] are associated with altered gut microbiota composition, along with compromised gut barrier function, which increases intestinal permeability, facilitates bacterial toxins into circulation, and enhances systemic inflammation [8,9]. Moreover, PN in type 2 diabetes patients is associated with altered microbiota signature [10,11]. In an HFD mouse model of obesity, we have shown that PN correlates with sciatic nerve lipidome and inflammation, which dietary reversal to SD rescued [12]. These findings suggest an intersection between obesity, diabetes, inflammation, and the microbiome on PN development. Indeed, switching from an SFA- to a MUFA-rich diet modulates gut microbiome composition [13] and inflammation [14,15], just as we have shown it ameliorates PN [4]. Altering the gut microbiome directly, such as by fecal microbiota transplantation (FMT) from lean donor mice to obese SFA-fed recipient mice alleviates neuropathic pain and sciatic nerve inflammation, effects that correlate with circulating levels of microbiome-derived short-chain fatty acid (SCFA) lipids [16].

However, the influence of dietary fatty acid composition on gut microbiome in the context of obesity-induced PN is understudied [16]. Herein, we postulated that an SFA- versus MUFA-rich diet or SD would impact gut microbiome composition and correlate with PN development. To assess causality, we also evaluated the impact of FMT from SFA- versus MUFA-rich diet or SD donor mice to lean recipient mice on metabolic and PN phenotypes. Finally, we examined the ability of FMT to modulate the expression of inflammatory markers in the colon and lipid transport proteins in the sciatic nerve.

## 2. Materials and Methods

### 2.1. Mouse Model and Study Design

We previously reported that mice fed an SFA-rich HFD (hereon referred to as SFA) develop obesity, prediabetes, and PN [3,4,12]. The current study uses the same model in two separate cohorts. In Cohort 1, which was previously published [4], 6-week-old male wild-type (WT) C57BL/6J mice (stock #000664, Jackson Laboratories, Bar Harbor, ME, USA) were placed on a 10-week regimen of either SD (D12450J comprising 10% kcal fat, Research Diets, New Brunswick, NJ, USA) or lard-based, SFA-rich HFD (D12492 comprising 60% kcal fat, Research Diets). Subsequently, a subset of SFA mice was switched to an 8-week regimen with fat calories replaced with MUFAs (hereon referred to as MUFA; D18043009 comprising 60% kcal fat derived from high MUFA sunflower oil, Research Diets). SD mice and remaining SFA mice stayed on their respective diets for the same 8-week duration. At the end of the study, ileum, cecum, and colon content, and fecal pellets from SD (*n* = 7), SFA (*n* = 4–5), and MUFA (*n* = 7) mice were banked at −80 °C freezers for the present study.

For Cohort 2, the FMT study, 4-week-old male WT C57BL/6J mice received an antibiotic cocktail (0.5 mg/mL amoxicillin, 2.5 mg/mL vancomycin, 0.5 mg/mL metronidazole, 0.025 mg/mL amphotericin B, 0.025 mg/mL streptomycin in drinking water) or control vehicle (autoclaved water) for 10 days. The antibiotic cocktail was optimized to deplete over 90% of gut microbiota without affecting the health or development of the mice [17]. The antibiotic cocktail was administrated in drinking water to prevent distress from gavage or injection at this young age. At approximately 6 weeks of age, a control group of mice, “no antibiotics, no FMT” remained on vehicle autoclaved water lacking antibiotics and did not receive FMT (*n* = 12 mice). FMT was prepared by homogenizing fecal pellets in a solution of phosphate-buffered saline supplemented with 0.05% of L-cysteine and passed through a 40 μm cell strainer [17]. The mice on antibiotics were divided into four groups (*n* = 12 mice per group) and all treatments occurred via oral gavage: (i) a “no FMT” sham procedure group of mice that remained on an antibiotic cocktail and received phosphate-buffered saline; (ii) a “SD FMT” group of mice that received FMT from donor SD mice, which were fed SD until 16 weeks of age; (iii) an “SFA FMT” group of mice that received FMT from donor SFA mice, which were fed SFA-rich, lard-based HFD until 16 weeks of age; (iv) a “MUFA FMT” group of mice that received FMT from donor MUFA mice, which were fed SFA HFD until 16 weeks of age followed by MUFA diet for 8 weeks. FMT recolonized the gut and was scheduled once weekly for 10 weeks until the mice were 16 weeks of age. During that time, mice were fed normal chow (5LOD, catalog no. 3005659-220, LabDiet, Oxford, MI, USA). At study end, mice were sacrificed by sodium pentobarbital (150 mg/kg, intraperitoneal injection; Vortech Pharmaceutical, Dearborn, MI, USA). Colons were dissected from under the cecum to the rectum while sciatic nerves were dissected from their origin at the sacral area through the popliteal fossa where it divides into two branches to be used for quantitative real-time PCR (qPCR) and Western blotting. The tissues were frozen and stored at −80 °C.

All animal protocols were approved by the University of Michigan Institutional Animal Care and Use Committee (protocol PRO00008115 for Cohort 1, approval period: 15 January 2018–15 January 2021; protocol PRO00010039 for Cohort 2, approval period: 28 January 2021–28 January 2024) and were performed in compliance with University guidelines, State and Federal regulations, and the standards of the National Institutes of Health, “Guide for the Care and Use of Laboratory Animals”.

### 2.2. Metabolic and Peripheral Neuropathy Phenotyping

For Cohort 1, mice were weighed at the end of the study along with glucose tolerance testing [4]. For Cohort 2, mice were weighed every two weeks, starting at 5 weeks until 15 weeks of age. Fasting blood glucose and glucose tolerance tests were performed at 5, 7, and 15 weeks of age, per our published protocols [3,4,12]. Briefly, for both cohorts, mice were fasted for 4 h, and fasting blood glucose was measured from a drop of tail blood using a glucometer (AlphaTrak, Zoetis, Parsippany, NJ, USA). Mice were then administered an intraperitoneal injection of glucose (1 g/kg body mass) in normal saline. A glucometer measured blood glucose levels from one drop of tail blood at baseline and at 15, 30, 60, and 120 min, following glucose injection.

PN phenotyping was performed in the study using protocols established by the Diabetes Complications Consortium (http://www.diacomp.org). Prior to sacrifice, large fiber PN was assessed by nerve conduction velocity (NCV) from sural sensory nerves and sciatic–tibial motor nerves, per our published protocols [3,4,12]. Briefly, isoflurane-anesthetized animals were placed under a heating lamp to hold their core temperature at 34 °C. Stainless steel needle electrodes (Natus Medical, Pleasanton, CA, USA) recorded sural sensory NCVs at the foot dorsum after antidromic supramaximal stimulation at the ankle. Sensory NCVs were calculated by dividing the distance by the sensory nerve action potential take-off latency. Sciatic–tibial motor NCVs were recorded at the foot dorsum after orthodromic supramaximal stimulation, first at the ankle followed by at the sciatic notch. Latencies were recorded for each location from the initial onset of the compound muscle action potential. Sciatic–tibial motor NCVs were calculated by subtracting the measured ankle distance from the measured notch distance and dividing by the difference in ankle and notch latencies. NCVs were represented as meters per second (m/s).

Small fiber PN was assessed by intraepidermal nerve fiber density (IENFD), per our published protocols [3,4,12]. Briefly, after mice were sacrificed in the study, plantar hind paw footpads were collected and fixed in Zamboni Fixative (catalog no. 1459A, Newcomer Supply, Middleton, WI, USA) for 2 h at 4 °C. The following day, footpads were rinsed in 30% sucrose solution in 0.1 M sodium phosphate buffer, cryoembedded, and sectioned to a 30 µm thickness. Immunohistochemistry was performed on sections using UCHL1/PGP9.5, a pan-axonal marker (1:2000; catalog no. 14730-1-AP, Proteintech, Rosemont, IL, USA), followed by donkey anti-rabbit IgG Alexa Fluor 488 secondary antibody (1:2000; catalog no. A-21206, Thermo Fisher Scientific, Waltham, MA, USA). Sections were imaged by confocal microscopy (Leica SP5, 20 × 1.2 objective, 3.3 µm optical sections, 1024 × 1024 pixels; Leica Microsystems, Deerfield, IL, USA). A blinded investigator counted fibers from three stacked images per mouse and represented IENFD as the number of fibers per millimeter (fiber/mm).

### 2.3. Microbiome Profiling and Analysis

Intestinal and fecal sample collection and microbiome analysis were performed using our published protocols [12,18]. Briefly, fecal samples were collected directly into a sterile Eppendorf tube before mice were sacrificed. After sacrifice, samples were collected under aseptic conditions from the ileum, cecum, and colon. Bacterial DNA was extracted by seeding intestinal and fecal samples into a PowerMag Glass Bead Plate supplied in the MagAttract PowerMicrobiome DNA/RNA Kit (catalog no. 27500-4-EP, Qiagen, Germantown, MD, USA) and epMotion 5075 liquid handling system. The V4 region of the bacterial 16S rRNA gene was sequenced on an Illumina MiSeq (Illumina, San Diego, CA, USA) using a standard protocol [19] at the University of Michigan Microbiome Core.

Raw sequencing reads were filtered, de-replicated, and de-noised, and the microbe taxonomy of the resulting amplicon sequence variants (ASVs) was assigned using the SILVA v138.1 database [20] after removing chimeras, all implemented in the microbial R package (https://cran.r-project.org/web/packages/microbial/microbial.pdf, accessed on 1 November 2024) with functionalities imported from the dada2 package [21]. Any ASVs SILVA could not identify at the phylum level were categorized as “unassigned”. ASVs were removed from downstream analysis if they were unassigned at the phylum level or were assigned, but present in fewer than three samples, using a prevalence threshold < number of samples × 0.05. Various metrics assessed sample alpha diversity using microbial R package. To evaluate beta diversity, a principal coordinate analysis based on Bray–Curtis dissimilarity metrics was conducted using the proportional normalized data. A heatmap of the log_10_-transformed microbiota abundance was constructed using the microbiomeutilities R package (https://microsud.github.io/microbiomeutilities/, accessed on 1 November 2024). Differential ASV abundance was evaluated by the DESeq2 package [22] using a significance threshold of *p*-value < 0.05. Pearson’s correlation between microbial abundance and sensory and motor NCVs was calculated and plotted using functions from the microbiomeSeq R package (https://github.com/umerijaz/microbiomeSeq, accessed on 1 November 2024).

### 2.4. Evaluation of Fatty Acid Transporters and Inflammatory Markers

mRNA was measured by qPCR, per our published protocols [23]. Briefly, mRNA was extracted from colon tissue using RNeasy Mini Kit (Qiagen, catalog no. 74104) and concentrated using Norgen RNA Clean-Up and Concentration kit (catalog no. 43200, Norgen Biotek, Thorold, ON, Canada) and reverse transcribed into cDNA by iScript (catalog no. 1708841, Bio-Rad, Hercules, CA, USA). qPCR reactions were prepared using *Gpr43-* (Taqman assay ID: Mm01176527_m1), *Il1β-* (Taqman assay ID: Mm00434228_m1), or *Tlr4*- (Mm00445273_m1) specific probes, forward and reverse primers with the cDNA template and PCR TaqMan Gene Expression master mix (catalog no. 4369016, Thermo Fisher Scientific). Reactions were subjected to a qPCR program on a StepOnePlus Real-Time PCR system (Applied Biosystems, Thermo Fischer Scientific). Control reactions using the 18S reference gene were run in tandem using a multiplex system with all probes of interest in the FAM channel and the control probe in the VIC channel. In sum, technical duplicates of biological triplicates were performed for each gene of interest. mRNA was quantified using the 2^−ΔΔCt^ method relative to the reference gene.

Protein was quantified by Western blot (WB), as per our published protocols [24]. Briefly, sciatic nerve samples were lysed in T-PER™ Tissue Protein Extraction Reagent (catalog no. 78510, Thermo Fisher Scientific) buffer supplemented by protease inhibitor (catalog no. 11836170001, MilliporeSigma, Rockville, MD, USA), protein quantified by Pierce protein assay (catalog no. 22662, Thermo Fisher Scientific), and 25 µg protein resolved by SDS-PAGE. FXR was detected using primary rabbit anti-mouse FXR (catalog no. 72105S, Cell Signaling Technologies, Danvers, MA, USA) and HSC70 reference protein using primary rat anti-mouse HSC70 antibody (catalog no. MA1-26078, Invitrogen, Thermo Fisher Scientific). Anti-rabbit (catalog no. 7074S, Cell Signaling Technologies) and anti-rat (catalog no. 7077S, Cell Signaling Technologies) secondary antibodies were used, and bands visualized using Clarity Max ECL Western blotting Substrates (catalog no. 1705062, Bio-Rad). Bands were visualized using the Bio-Rad UV Gel Documentation System (Bio-Rad). The visualized bands were quantified using the ImageJ software 1.54g [25].

### 2.5. Statistical Analyses

Statistical analyses of all data sets were performed using Prism (v10.4.1; GraphPad, San Diego, CA, USA) or R (v 4.4.0, R Core Team, Vienna, Austria) [26]. The sample size for the first cohort was based on literature with the minimum number of mice selected to investigate the effect of diet on PN. For the FMT cohort, we used the G*Power v3.1.9.7 software [27,28] to calculate sample size based on empirical effect size of 0.5, alpha = 0.05, and 80% power [29]. We selected “F tests” from the test family and “ANOVA” for the statistical test. The suggested sample size was 55 (*n* = 11/group). For all comparisons between multiple groups, one-way ANOVA with Tukey’s post hoc test for multiple comparisons was used if data were normally distributed by Shapiro–Wilk or Kolmogorov–Smirnov tests. Otherwise, Kruskal–Wallis with Dunn’s post hoc test for multiple comparisons was implemented, as indicated. Most data are represented as mean ± standard deviation. Statistical significance was set at *p* < 0.05.

## 3. Results

### 3.1. Monounsaturated Fatty Acid-Rich Diet Protects Against Saturated Fatty Acid-Rich Diet-Induced Peripheral Neuropathy in Mice

**Figure 1:**
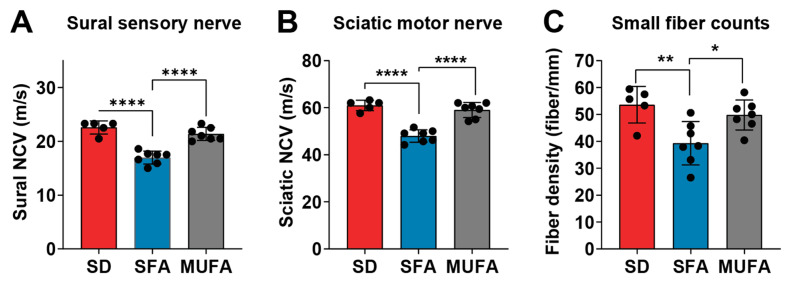
MUFA diet protects against SFA-induced PN in mice. WT mice aged 6 weeks received a standard diet (SD, red) or saturated fatty acid (SFA)-rich diet (SFA, blue) for 10 weeks. Half the SFA mice were then placed on a diet rich in monounsaturated fatty acids (MUFA, gray) for 8 weeks while the remaining mice stayed on their respective diets. Terminal assessment of large fiber neuropathy by (**A**) sural sensory and (**B**) sciatic motor nerve conduction velocity (NCV). (**C**) Terminal assessment of small fiber neuropathy by intraepidermal nerve fiber density. Black circles represent individual animals. All data presented as mean ± standard deviation; *n* = 5–7 mice per group; * *p* < 0.05, ** *p* < 0.01, **** *p* < 0.0001 for SD vs. SFA; SFA vs. MUFA; one-way ANOVA with Tukey’s multiple comparisons test. Figure adapted ref. [4].

We previously reported the effects of different fatty acid-rich diets on nerve function [4]. In this published study, we placed 6-week-old male WT mice on a 10-week regimen of either SD or SFA-rich lard-based HFD (hereon referred to as SFA). Subsequently, in a subset of SFA mice, the lard-based diet was replaced with a MUFA-rich diet (hereon referred to as MUFA) for an additional 8 weeks, while the remaining mice stayed on their respective diets. SFA mice developed obesity, impaired glucose tolerance, and high-fat mass, along with PN, manifested by slower NCV and lower IENFD (Figure 1, adapted from ref. [4]). On the other hand, PN was rescued in SFA mice that were switched to the MUFA diet for 8 weeks, reflected by restored NCVs and IENFDs to values commensurate with SD mice, although weight, glucose tolerance, and fat mass were not rescued. These findings suggested improved PN in MUFA mice independent of improved systemic metabolism.

### 3.2. Different Fatty Acid-Rich Diets Associate with Distinct Gut Microbiome Community Structure and Peripheral Neuropathy

**Figure 2:**
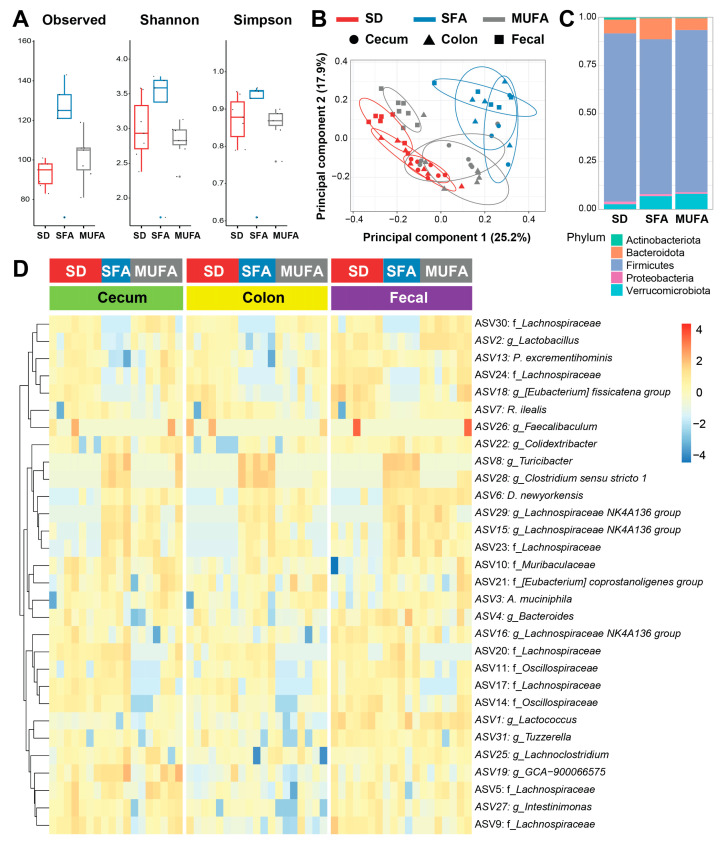
Different fatty acid-rich diets associated with distinct gut microbiome community structures. (**A**) Intra-group microbial diversity assessed by alpha diversity in fecal samples using observed counts and Shannon and Simpson indices from standard diet (SD, red), saturated fatty acid-rich diet (SFA, blue), and monounsaturated fatty acid-rich diet (MUFA, gray) mice. Data in box plots represented with horizontal line for median, box for first and third quartiles, and whiskers for minimum and maximum values. (**B**) Inter-group microbial diversity assessed by beta diversity. Principal coordinate analysis based on ASV clustering of gut microbiome from cecum (circle), colon (triangle), and fecal (square) samples in SD (red), SFA (blue), and MUFA (gray) mice. Ellipses comprise 85% of samples. (**C**) Stacked bar plot of relative abundance of the most abundant gut microbiome phyla in fecal samples from SD, SFA, and MUFA mice. (**D**) Heatmap clustering based on log_10_-transformed ASV genera abundance in gut microbiome from cecum (green), colon (yellow), and fecal (purple) samples in SD (red), SFA (blue), and MUFA (gray) mice. Legend indicates lower (blue) to higher (red) genera (g_) or family (f_) abundance. Data from SD (*n* = 7), SFA (*n* = 4–5), and MUFA (*n* = 7) cecum, colon, and fecal microbial samples.

We next addressed whether the differential effects of an SFA- versus MUFA-rich diet on PN are linked to gut microbiome changes. First, we assessed alpha diversity, a measure of within-sample microbial diversity. We found site-specific alpha diversity in cecum, colon, and fecal samples that were significantly higher than in ileum samples (Supplementary Figure S1A), as aligned with the literature [30]. Since ASV counts in the ileum were low, they were only analyzed for alpha diversity. We found that alpha diversity in the cecum, by observed counts, was significantly higher in the SFA-rich diet versus SD mice (Supplementary Figure S1B). MUFA alpha diversity levels were commensurate with SD mice. The pattern of higher alpha diversity in the SFA versus SD and MUFA microbiome persisted by the Shannon and Simpson indices, albeit non-significantly. Although alpha diversity was higher in SFA versus SD and MUFA colon (Supplementary Figure S1C) and fecal samples (Figure 2A), these differences were not significant.

Next, we examined beta diversity, a measure of inter-group gut diversity, which we presented by principal coordinate analysis plots. We found that SD, SFA, and MUFA samples fell into distinct clusters, in all niches, cecum, colon, or fecal, indicating between group differences in gut microbial composition (Figure 2B, Supplementary Figure S2). Interestingly, the MUFA samples tended to cluster between the SD and SFA clusters. Comparing the relative abundance of the major bacterial phyla, cecum samples showed a significant decrease in Proteobacteria in SFA versus SD mice (Supplementary Figure S3A,B), without significant differences by diet in Actinobacteriota, Bacteroidota, Firmicutes, and Verrucomicrobiota. Similarly, there were only minor diet-induced phyla differences in the colon, with only higher Verrucomicrobiota counts in MUFA versus SD colon samples (Supplementary Figure S3C,D), and no phyla differences in fecal samples (Figure 2C, Supplementary Figure S3E). Overall, there were few phyla structure differences by diet and site.

**Figure 3:**
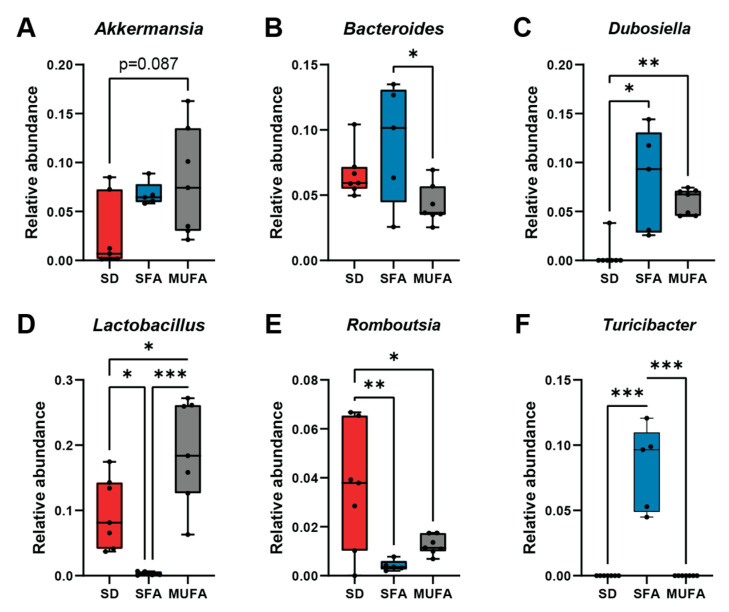
Different fatty acid-rich diets associated with differential fecal bacterial genera abundance. (**A**–**F**) Relative abundance of the most and differentially abundant genera in fecal microbial samples from the standard diet (SD, red, *n* = 7), saturated fatty acid-rich diet (SFA, blue, *n* = 5), and monounsaturated fatty acid-rich diet (MUFA, gray, *n* = 7) mice. Data in box plots represented with horizontal line for median, box for first and third quartiles, and whiskers for minimum and maximum values. Black circles represent individual animals. * *p* < 0.05, ** *p* < 0.01, *** *p* < 0.001; Kruskal–Wallis with Dunn’s multiple comparisons test for panels (**A**, **C**, and **F**); one-way ANOVA with Tukey’s multiple comparisons test for panels (**B**, **D**, and **E**).

Moving down the taxonomic structure to the genera level, unsupervised clustering by microbe abundance distinctly separated SD from SFA from MUFA mice across all sites, cecum, colon, and fecal (Figure 2D). *Bacteroides*, *Dubosiella*, and *Turicibacter* were more abundant while *Lactobacillus* and *Romboutsia* were less abundant in SFA versus MUFA and/or SD fecal samples (Figure 3; Supplementary Table S1). Lower *Lactobacillus* and higher *Dubosiella* and *Turicibacter* abundance were similarly present in SFA versus MUFA and/or SD cecum and/or colon samples, although the pattern of *Akkermansia* and *Romboutsia* abundance by diet varied in the colon compared to fecal samples (Supplementary Figure S4; Supplementary Table S1). Overall, MUFA reversed some, but not all, SFA-induced changes in the most abundant genera.

**Figure 4:**
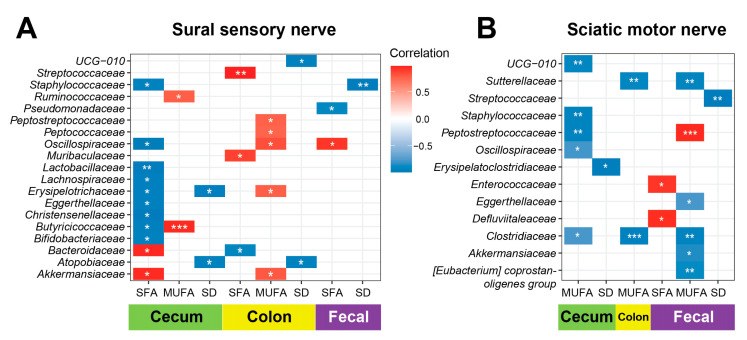
Sural and sciatic nerve conduction velocities correlate with distinct microbiota families in different fatty acid-rich diet mouse groups. Heatmap of Pearson correlation analysis of (**A**) sural sensory and (**B**) sciatic motor nerve conduction velocities to family taxa in cecum (green), colon (yellow), and fecal (purple) microbial samples from standard diet (SD, *n* = 7), saturated fatty acid-rich diet (SFA, *n* = 4–5), and monounsaturated fatty acid-rich (MUFA, gray, *n* = 7) mice. Legend indicates negative (blue) and positive (red) correlations. Unadjusted *p*-values, * *p* < 0.05, ** *p* < 0.01, *** *p* < 0.001; Pearson correlation analysis.

Next, we examined the correlation of PN phenotypes by NCVs to microbiome families (Figure 4). Sural sensory NCV most significantly correlated positively to *Butyricicoccaceae* in MUFA cecum and *Streptococcaceae* in SFA colon and negatively to *Staphylococcaceae* in SD fecal samples and *Lactobacillaceae* in SFA cecum. Most correlations of sural sensory NCV to microbiota arose in SFA samples, especially in the cecum. Sciatic motor NCV most significantly correlated positively to *Peptostreptococcaceae* in MUFA fecal samples and negatively to *Clostridiaceae* in MUFA colon. Most correlations of sciatic motor NCV to microbiota arose in MUFA samples.

### 3.3. Saturated and Monounsaturated Fatty Acid-Rich Diet-Derived Fecal Microbiota Transplantation Does Not Affect Metabolic Phenotypes nor Substantially Impact Peripheral Neuropathy in Recipient Mice

**Figure 5:**
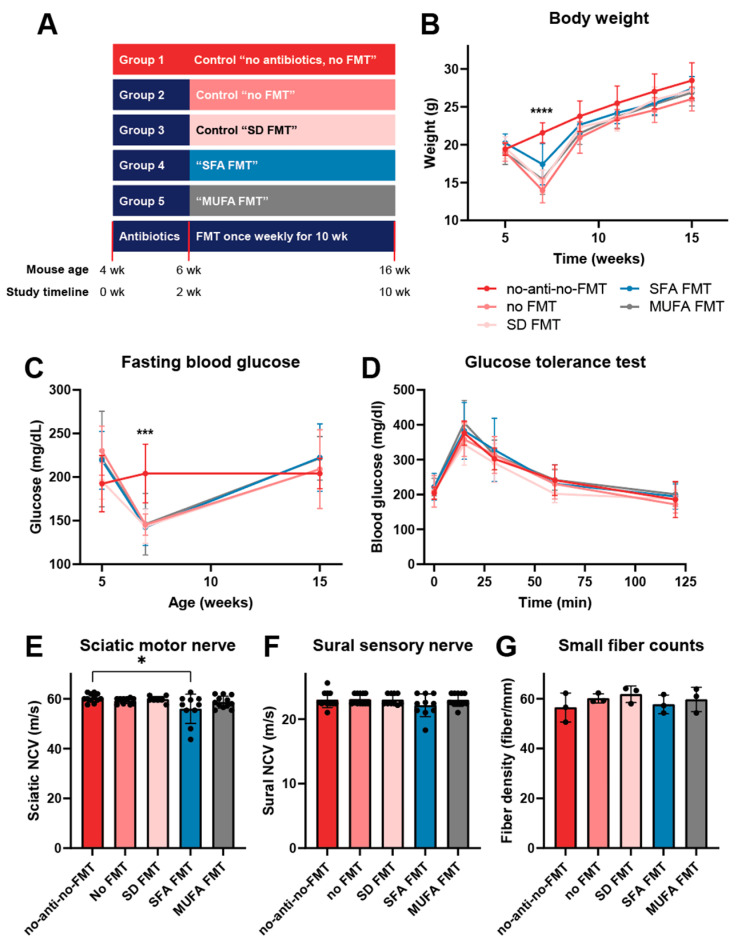
Different FMT transplants do not substantially affect metabolic and PN phenotypes in recipient mice. (**A**) FMT study design and mouse groups. Longitudinal (**B**) body weight and (**C**) fasting blood glucose levels for all mouse groups at study start (aged 5 weeks), after microbiota depletion (aged 7 weeks), and towards study end (aged 15 weeks). (**D**) Blood glucose levels following glucose tolerance test for all mouse groups towards study end (aged 15 weeks). Data presented as mean ± standard deviation; *n* = 11–12 mice per group; *** *p* < 0.001, **** *p* < 0.0001 for “no antibiotics, no FMT” versus other groups, otherwise few other statistically significant between-group differences; two-way ANOVA with Dunnett’s multiple comparisons. Terminal assessment of large fiber neuropathy by (**E**) sciatic motor and (**F**) sural sensory nerve conduction velocity (NVC). Data presented as mean ± standard deviation; *n* = 10–12 mice per group; * *p* < 0.05 for “no antibiotics, no FMT” versus SFA FMT, Kruskal–Wallis with Dunn’s multiple comparisons. (**G**) Terminal assessment of small fiber neuropathy by intraepidermal nerve fiber density. Black circles represent individual animals. Data presented as mean ± standard deviation; *n* = 3 mice per group; Kruskal–Wallis with Dunn’s multiple comparisons. No-anti-no-FMT, no antibiotics, no FMT; no FMT, no fecal microbiota transplant; SD FMT, standard diet FMT; SFA FMT, saturated fatty acid FMT; MUFA FMT, monounsaturated fatty acid FMT.

To dissect the role of microbiota on PN development and examine possible causality, we designed an experiment of WT mice fed SD that received FMT from various donor mice. Recipient mice were first depleted of their endogenous microbiota by an antibiotic regimen and then inoculated with FMT from donor mice fed specific diets (Figure 5A). SFA FMT and MUFA FMT mice received FMT from donor SFA and MUFA mice, respectively. The SFA FMT group was designed to evaluate whether the gut microbiota that flourishes in donor mice on the SFA diet induce PN in recipient mice; on the other hand, microbiota from donor mice on a protective MUFA diet are not expected to cause PN. These experimental groups were compared to several control groups, control mice fed SD that were never depleted of their endogenous microbiota and SD-fed mice that were depleted of their endogenous but did not receive FMT or received SD FMT.

All groups had the same starting weight at the age of 5 weeks (Figure 5B); however, antibiotic-treated mice initially lost weight (aged 7 weeks) before they regained it towards the end of the study (aged 15 weeks). Meanwhile, the group that never received antibiotics gained weight steadily and differed from all other groups at 7 weeks of age. Towards the end of the study, all mice had the same ending weight at the age of 15 weeks. A similar pattern was observed with fasting blood glucose (FBG) levels; at the start of the study, all groups had similar FBG levels, which dropped slightly in all antibiotic-treated groups at 7 weeks of age but not in the group that did not receive antibiotics (Figure 5C). Towards the end of the study, all mouse groups again manifested the same FBG levels, reflected by similar results in the glucose tolerance test at 15 weeks of age (Figure 5D).

Finally, we assessed the PN phenotype of all mouse cohorts using NCV for large fiber function and IENFD for small fiber structure. Since antibiotic treatment initially altered the metabolic profile of mice, all comparisons in PN metrics were performed against the “no antibiotics, no FMT” control group. We found that SFA FMT significantly reduced motor NCVs, although only within a small margin of significance, with *p*-value < 0.05 (Figure 5E). However, there were no significant differences between the “no antibiotics, no FMT” mice to any other group in either sensory NCV (Figure 5F) or IENFD (Figure 5G).

### 3.4. Saturated and Monounsaturated Fatty Acid-Rich Diet-Derived Fecal Microbiota Transplantation Affects Inflammatory Gene Expression in Colon but Not Fatty Acid Transporter Expression in Colon or Sciatic Nerve of Recipient Mice

**Figure 6:**
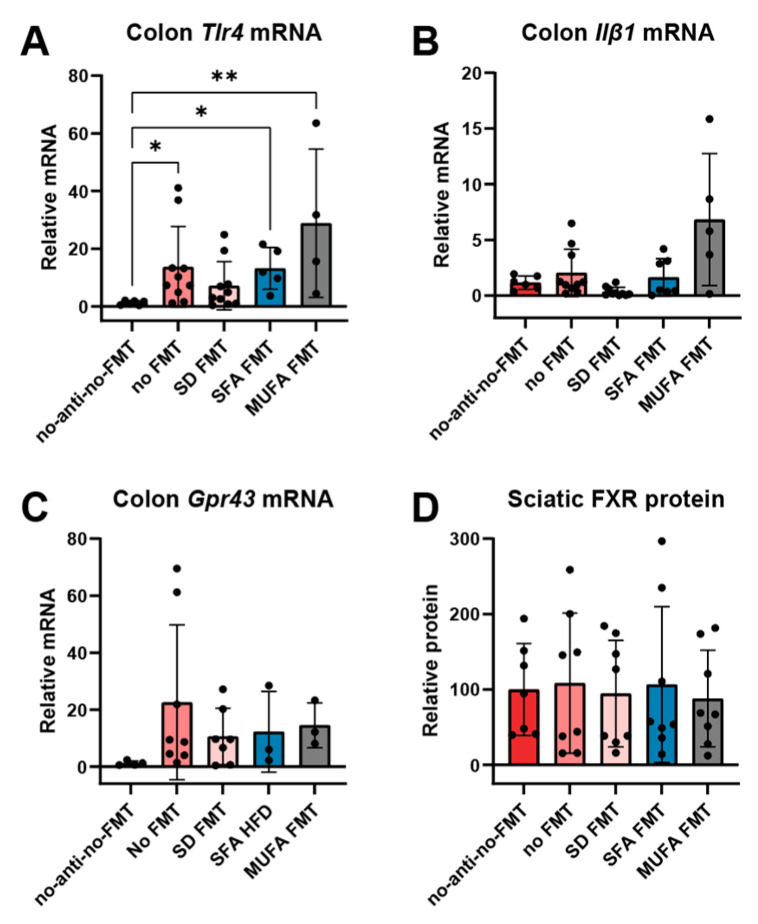
MUFA FMT transplant affects inflammatory gene expression in colon but not fatty acid transporter expression in colon or sciatic nerve of recipient mice. Terminal (**A**) *Tlr4*, (**B**) *Il1β*, and (**C**) *Gpr43* mRNA expression by qPCR of colon tissue from all mouse groups. mRNA calculated against 18S reference gene and expressed relative to the “no antibiotics, no FMT” group. (**D**) Terminal FXR protein expression by Western blot of sciatic nerve from all mouse groups. Protein calculated against HSC70 loading control and expressed relative to the “no antibiotics, no FMT group”. Black circles represent individual animals. *n* = 3–10 mice per group; * *p* < 0.05, ** *p* < 0.01 for “no antibiotics; no FMT” versus other groups; Kruskal–Wallis with Dunn’s multiple comparisons. No-anti-no-FMT, no antibiotics, no FMT; no FMT, no fecal microbiota transplant; SD FMT, standard diet FMT; SFA FMT, saturated fatty acid FMT; MUFA FMT, monounsaturated fatty acid FMT.

Inflammation is a pivotal pathway in mediating the effects of microbiota. Therefore, we investigated mRNA expression of both interleukin-1β (*Il1β*) and Toll-like receptor 4 (*Tlr4*) in colon tissues. Both *Il1β* [31] and *Tlr4* [32] elicit favorable inflammatory reactions to preserve intestinal barrier integrity and promote repair. We found that SFA and MUFA FMT upregulated *Tlr4* (Figure 6A) but not *Il1β* (Figure 6B) mRNA expression versus the “no antibiotics, no FMT” mice. The “no FMT” group, i.e., received antibiotics but not FMT, also increased *Tlr4* expression. We also investigated the effect of different FMT transplants on the expression of two key fatty acid receptors, G protein-coupled receptor 43 (GPR43) (also known as free fatty acid receptor 2), and farnesoid X-activated receptor (FXR). GPR43 mediates microbial action of SCFAs on inflammation [33], and is key in the gut–brain axis [34]. In our study, there were no significant differences in *Gpr43* mRNA expression levels in colon tissue (Figure 6C). FXR is also activated by microbiota and their metabolites [35] and is linked to the sensation of neuropathic pain via expression in the spinal cord [36]. Therefore, we examined FXR protein expression levels in the sciatic nerve, but found no significant differences (Figure 6D).

## 4. Discussion

Emerging evidence indicates that microbiome changes contribute to nervous system health, indicating a possible gut–peripheral nerve connection [5]. We previously demonstrated that a MUFA-rich diet reverses SFA-induced PN [4], which the literature suggests occurs in tandem with microbiome restructuring [13]. Moreover, metabolically acquired PN is characterized by a distinct microbiome structure [10,11]. In obese mice with prediabetes, we identified PN correlates to specific microbiota, also linked to sciatic nerve lipid species and inflammatory changes [12]. In sum, complex interrelationships between obesity, diabetes, inflammation, and the microbiome are associated with PN development. Herein, we investigated the connection between SFA- and MUFA-rich diets to the microbiome in the framework of obesity-induced PN, which, to our knowledge, has not been reported. We found that SFA promoted changes in microbiome community structure versus lean SD microbiome in colon, cecum, and fecal sites, which MUFA partially reversed, albeit remaining distinct from SD microbiota profiles. Sciatic and sensory NCVs correlated with several microbial families across SD, SFA, and MUFA mice and gastrointestinal sites. In FMT experiments, neither SFA nor MUFA FMT affected metabolic phenotypes overall in recipient mice although SFA FMT marginally lowered motor NCV at the end of the study.

This study was prompted by our earlier finding that a dietary change in fatty acid composition from SFA to MUFA reverses PN in mice, without concurrent weight-loss [4]. Switching rats from SFA to diets rich in polyunsaturated fatty acids (PUFAs) similarly improves PN independent of changes in weight [37]. There are few human studies, and the impact of SFA- versus MUFA- and PUFA-rich diets on PN in diabetic and/or obese patients remains unclear. However, observational population-based reports suggest PUFA intake correlates to improved peripheral nerve function [38,39]. In participants with type 2 diabetes, adherence to a Mediterranean diet, a diet rich in MUFAs and PUFAs, is linked to improved PN, independent of weight [40]. Intervention with daily PUFAs for 3 months lowered pain symptoms linked to diabetic PN and plasma inflammatory and oxidative stress biomarkers, but not weight, in participants with type 2 diabetes and obesity [41]. Although large, randomized controlled trials are needed, the emerging literature suggests that dietary fatty acid composition may impact PN onset and development.

We next sought to assess the influence of SFA- and MUFA-rich diets on microbiome structure and possible correlation to PN. Excessive intake of foods rich in sugars and SFAs prompts changes in microbiome composition [42,43]. In our comparison of microbiome structure by site, we found alpha diversity was higher in cecum, colon, and fecal versus ileum samples, as reported in the literature [30]. By dietary fatty acid composition, we found alpha diversity was higher in SFA versus SD mice in cecum only, with no differences in colon or fecal samples. Our alpha diversity results are at odds with reports that obesity and SFA-rich diets lower alpha diversity [13], at least in fecal samples; however, the literature is not fully concordant and some studies note no difference from fatty acid composition on alpha diversity [13,44]. Although a richer microbiome is usually considered healthy, it is possible, rather, that loss or gain of key ‘core’ microbiota linked to health is more critical than overall richness [45]. Our examination by beta diversity clustered samples into distinct groups, with the MUFA group clustering between the SD and SFA groups. Microbiome profiles of humans with high versus low SFA or MUFA intake similarly separate into clusters [46], although we observed stronger separation in mice microbiota, possibly because humans have more varied diets.

In our analysis of the relative abundance of the major bacterial phyla, we observed few differences by diet across sites. Although, counter to our results, many studies report that SFA increases Firmicutes and decreases Bacteroidota abundance, both in humans and mice, the literature is not fully concordant [7,14]. SFA increased Proteobacteria in obese/overweight humans compared to unsaturated fatty acid intake [44], whereas we noted a decrease in our mice. We also examined differential levels in the most abundant genera by fatty acid intake; MUFA profiles tended to fall between SD and SFA profiles, and MUFA reversed some, but not all, SFA-induced changes in genera abundance. *Lactobacillus*, a probiotic [47], was lower in SFA versus MUFA and SD, as broadly aligned with literature, with reduced *Lactobacillus intestinalis* in SFA-induced obesity [14,48] and higher *Lactobacillus* following Mediterranean diet intake [49]. *Bacteroides* was elevated in SFA versus MUFA fecal samples; high fat intake [43,50] and Mediterranean diets [49] increase levels of this genus, which metabolizes complex molecules, but our results suggest that SFA-rich diets may exert more profound effects on *Bacteroides* levels than MUFA-rich diets. One interesting candidate is *Akkermansia*, specifically the *Akkermansia muciniphila* species, which is linked to poor metabolic health, such as obesity and diabetes [51]. MUFA significantly increased *Akkermansia* abundance relative to SD colon, aligned with some literature reports [52].

Overall, for phyla and specific genera abundance linked to fatty acid intake, there are discrepancies across the literature between mouse and human reports as well as within mouse studies. Findings may vary in mice versus humans due to intrinsic differences in gastrointestinal tract [53]. Fatty acid intake duration, diet composition, i.e., precisely formulated chow in mice versus diverse human diets, and natural gut composition variation in studied populations can introduce discrepancies across studies [53]. Distinct microbiota colonize various portions of the gastrointestinal tract [30,47], and sampling location may vary by study. Moreover, within the same genus, different specific species may exert beneficial or harmful effects [54]; however, segregating ASV counts at the species level did not have enough power to detect differential abundance.

Our primary interest was PN and possible mediation by gut microbiota changes from dietary fatty acids. The human gut may impact PN and neuropathic pain through various potential mechanisms [5]. Harmful microbiota can compromise the gut barrier, facilitating bacterial toxin leakage into circulation, enhancing inflammation [8,9], which may contribute to PN. Furthermore, bacterial toxins can activate nociceptors, evoking pain sensations [55]. Conversely, beneficial microbiota-derived metabolites, such as SCFAs, can promote immune tolerance and, consequently, alleviate inflammatory processes linked to PN and pain [16]. Healthful microbiota may also ameliorate or correlate with improved systemic metabolism and inflammation [51], thereby mitigating PN risks.

Thus, given our focus on PN, we first examined sural and motor NCV correlations to microbiota families. In MUFA cecum, sural sensory NCV correlated positively to *Butyricicoccaceae*, a family that contains the butyrate-producing genus *Butyricicoccus* [56], and may represent a microbiome-SCFA-immune-mediated neuroprotective mechanism from MUFA. By contrast, in SFA colon and cecum, sural sensory NCV correlated positively to *Streptococcaceae* and negatively to *Lactobacillaceae*, respectively, both families that contain beneficial and pathogenic genera. In SD fecal samples, sural sensory NCV correlated negatively to *Staphylococcaceae*, encompassing the *Staphylococcus* genus, notable for several pathogenic members. In particular, *S. aureus* produces a toxin that evokes spontaneous pain, including mechanical and thermal hyperalgesia [57]. Thus, a negative correlation in SD mice could be favorable to PN phenotypes. In MUFA fecal and colon samples, sciatic motor NCV correlated positively to *Peptostreptococcaceae*, a family of fermentative bacteria [58], and negatively to *Clostridiaceae*, notably containing the *Clostridium* genus, which includes species that produce botulinum toxin, a potentially useful treatment approach for neuropathic pain [59]. Although several potentially interesting relationships emerged, overall, however, correlation of NCV to families, rather than genera, limited insight.

Human studies implicate distinct microbiome profiles in type 2 diabetes patients with PN [10,11] and pain [60], suggesting that modulating the microbiome by FMT may constitute a strategy for modifying gut–nerve interactions. We found SFA FMT to SD recipient mice lowered motor NCV, a measure of large fiber PN, although it did not impact sensory NCV nor IENFD, a small fiber measure. Despite these findings, we propose that the full impact of SFA FMT on PN remains unclear for two reasons. First, the margin of significance we observed for large fiber motor improvement was narrow and, secondly, it is atypical to preserve small fiber function in PN with concurrent large fiber dysfunction [61]; therefore, future studies are required to clarify the impact of SFA microbiome on causality in PN. As expected from a protective diet against PN [4], we noted that MUFA FMT did not induce PN by any measured metric. Prior studies support microbiota modulation of PN. FMT from donor patients with diabetes and PN aggravates PN in recipient *db*/*db* mice, as assessed by sciatic NCV, IENFD, and thermal and mechanical sensitivity, versus FMT from healthy participants or patients with diabetes but no PN [11]. Extending FMT from healthy donors to human recipients with PN similarly improves neuropathy by the Toronto Clinical Scoring System relative to placebo [11]. Another FMT intervention from lean donor mice to obese SFA recipient mice alleviates hypersensitivity in tandem with sciatic nerve inflammation [16].

Thus, interventional studies suggest possible causal microbiome roles in PN, potentially by modulating systemic metabolism or, alternatively, directly through gut–nerve interactions, e.g., immune modulation [8,9,16,51]. However, in our paradigm, SFA FMT did not worsen metabolism in recipient SD mice. Moreover, although SFA FMT increased colon expression of *Tlr4*, this effect was not specific, and MUFA FMT as well as the “no FMT” group experienced the same, possibly arising as a lingering effect from antibiotic treatment [62]. Therefore, although there may be a moderate effect of SFA FMT to induce PN, the mechanism remains unknown and, as stated above, further investigation is needed.

To our knowledge, this is the first study to evaluate the impact of SFA- and MUFA-enriched diets on PN and had several strengths First, we analyzed gut microbiota from several gastrointestinal niches, spanning colon, cecum, and fecal samples. Second, our FMT experiments were well-controlled to examine the possible causation of SFA gut microbiome on PN phenotypes. We also used objective small and large fiber measures to comprehensively assess PN phenotypes.

Nevertheless, our study also had weaknesses. First, our microbiome analyses were only cross-sectional, but the gut community is highly variable over time, even in the same organism [63]. Second, we only used male mice; however, there are sex differences in the gut microbiome–brain (nervous system) axis that warrant investigation with female mice as well [64]. Third, in our analysis of microbiome differences by diets, we focused on the most abundant genera, although less abundant genera could potentially and disproportionately affect health outcomes, and may be of greater interest. Fourth, our correlations in NCVs to microbiome were only to family taxa and were only nominally significant due to limited sample size; therefore, larger studies with more granular genus- or species-level information are needed to draw deeper insight. Additionally, since we did not include an antiviral in our antibiotic cocktail, our study only examined the contribution of bacteria from the microbiome on PN, although there is a very small nascent literature on the virome in type 2 diabetes [65] and diabetic complications, e.g., nephropathy [66]. Fifth, our current experiments examined the possibility that potentially harmful gut microbiome-derived metabolites induce PN, e.g., by induction of inflammation. It is alternatively possible that protective microbiome metabolites, such as SCFAs, prevent obesity- and/or SFA-induced PN. In this paradigm, FMT from donor SD mice to obese recipient mice would alleviate SFA-induced PN, and this is a future avenue of study. Lastly, endogenous expression of our select targets in colon and sciatic tissue was low leading to substantial variability, and was limited to a few targets only, which lacks the scope of a system-wide approach, such as transcriptomics. However, most of these weaknesses are inherent to research on the complex microbiome.

## 5. Conclusions

Overall, we found SFA-induced changes in microbiome community structure, which MUFA partly reversed, reflected in genera abundance. However, the MUFA microbiome community remained distinct from that of SD-fed mice. NCV metrics correlated with several microbial families, some containing genera with feasible mechanisms of action for microbiome-mediated effects on PN. However, neither SFA nor MUFA FMT improved metabolic phenotypes although SFA FMT marginally slowed motor NCV in recipient mice. Future investigations should examine alternative SFA-mediated mechanisms of nerve injury, such as inflammatory processes [67], or insulin insensitivity [68], as well as MUFA-mediated protective mechanisms, such as, potentially, through SCFAs.

## Data Availability

The original data presented in the study are openly available in Sequence Read Archive at https://dataview.ncbi.nlm.nih.gov/object/PRJNA1119514?reviewer=4s4ndc43vqdhf726oq1lfe80nt, BioProject: PRJNA1119514.

## Supplementary Materials

The following supporting information can be downloaded at https://www.mdpi.com/article/10.3390/nu17040737/s1. Figure S1: Alpha diversity by sites and fatty acid-rich diets; Figure S2: Beta diversity by sites and fatty acid-rich diets; Figure S3: Phyla abundance by sites and fatty acid-rich diets; Figure S4: Different fatty acid-rich diets associate with differential cecum and colon bacterial genera abundance; Table S1: Differential genera abundance in by site and fatty acid-rich diets.
