# Supplementary material for: Dietary Fatty Acid Composition Alters Gut Microbiome in Mice with Obesity-Induced Peripheral Neuropathy
Source: Nutrients. 2025 Feb 19;17(4):737. doi: 10.3390/nu17040737 (PMC11858455; doi:10.3390/nu17040737)
Supplement: Supplementary file 1 [file nutrients-17-00737-s001.zip › nutrients-3468971-supplementary.pdf]

Supplementary Information for

Dietary fatty acid composition alters gut microbiome in mice with obesity-induced peripheral neuropathy

Mohamed H. Noureldein,[1,2] Amy E. Rumora,[3] Samuel J. Teener,[1,2] Diana M. Rigan,[1,2] John M. Hayes,[1,2] Faye E. Mendelson, Andrew D. Carter,[1,2] Whitney G. Rubin,[1,2] Masha G. Savelieff,[4] Eva L. Feldman[1,2,*]

[1]Department of Neurology, University of Michigan, Ann Arbor, MI

[2]NeuroNetwork for Emerging Therapies, University of Michigan, Ann Arbor, MI

[3]Department of Neurology, Columbia University, New York, NY

[4]Department of Biomedical Sciences, University of North Dakota, Grand Forks, ND

*Corresponding author

Eva L. Feldman, MD, PhD

109 Zina Pitcher Place

Ann Arbor, MI 48109-2200

Phone: 734-936-8586

Fax: 734-936-5185

E-mail: efeldman@umich.edu

Supplementary Figure S1. Alpha diversity by sites and fatty acid-rich diets. Intra-group microbial diversity assessed by alpha diversity using observed counts and Shannon and Simpson indices by (**A**) sites [cecum (green), colon (yellow), fecal (purple), ileum (grey)] and from (**B**) cecum and (**C**) colon samples in standard diet (SD, red, n=7), saturated fatty acid-rich diet (SFA, blue, n=4-5), and monounsaturated fatty acid-rich diet (MUFA, grey, n=7) mice. Data in box plots represented with horizontal line for median, box for first and third quartiles, and whiskers for minimum and maximum values. *p<0.05, **p<0.01, Wilcoxon test.

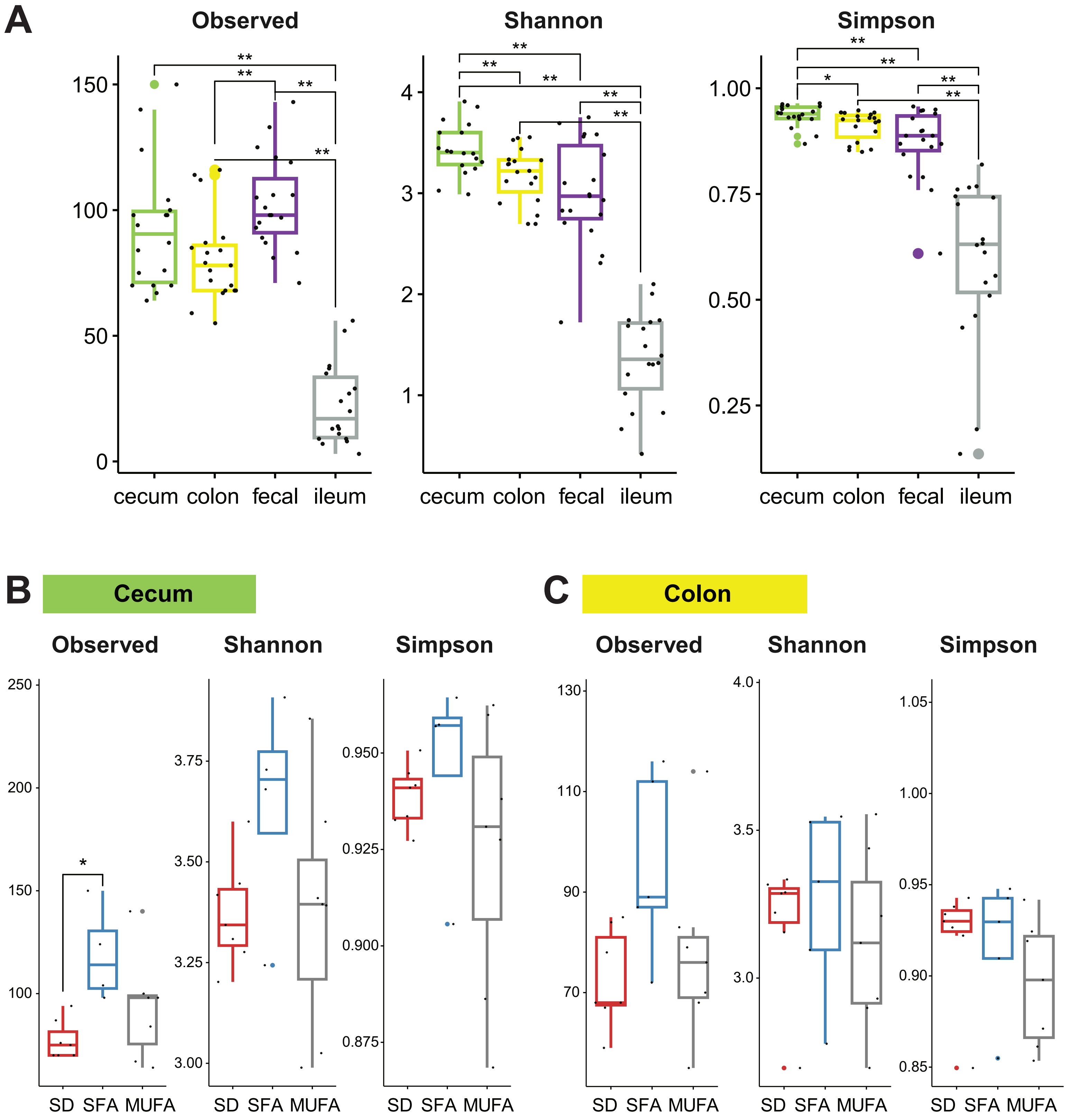

Supplementary Figure S2. Beta diversity by sites and fatty acid-rich diets. Inter-group microbial diversity assessed by beta diversity. Principal coordinate analysis based on ASV clustering of gut microbiome from (**A**) cecum (circle), (**B**) colon (triangle), and (**C**) fecal (square) samples in SD (red, n=7), SFA (blue, n=4-5), and MUFA (grey, n=7) mice. Ellipses comprise 85% of samples.

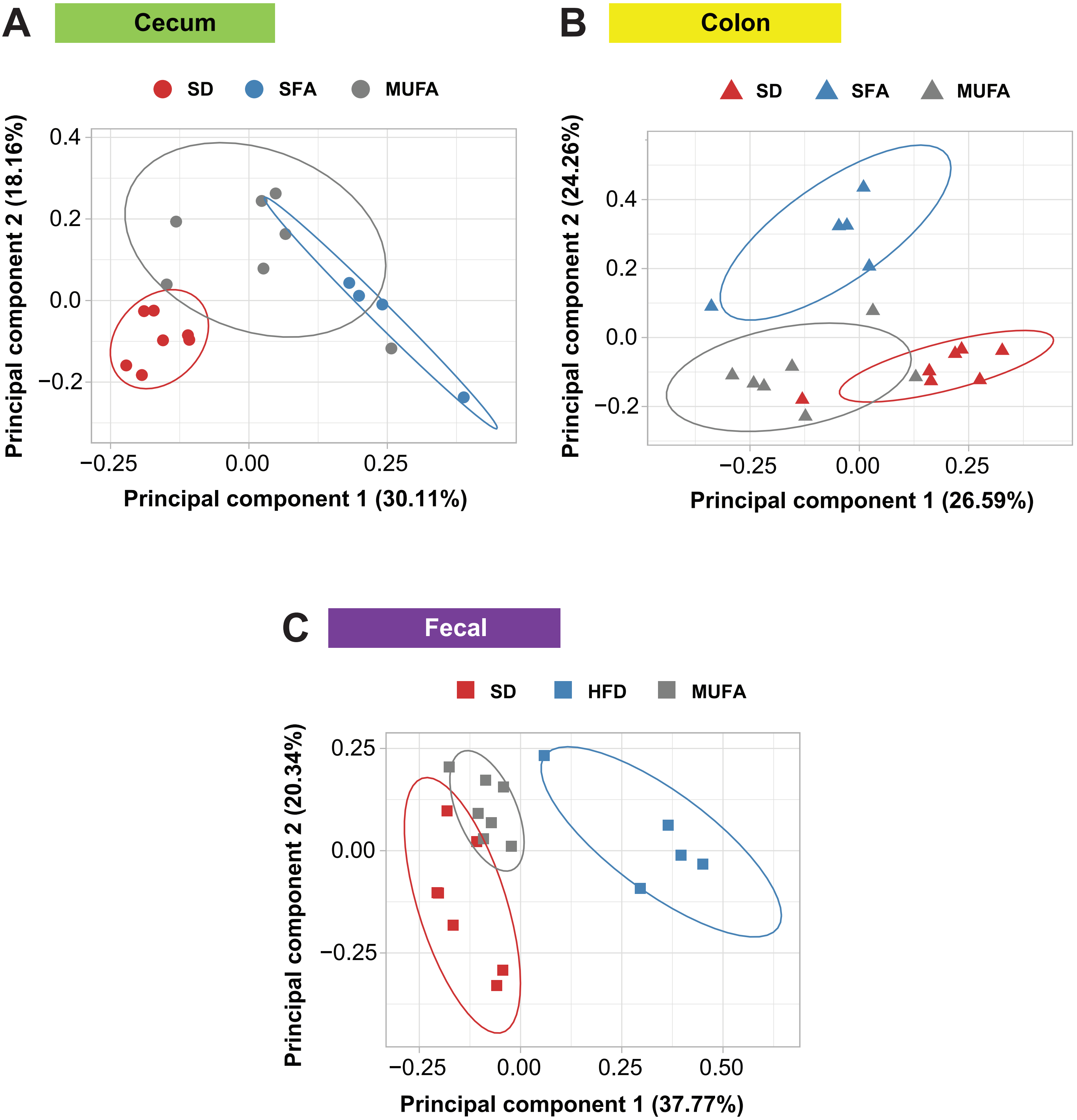

Supplementary Figure S3. Phyla abundance by sites and fatty acid-rich diets. (**A**) Stacked bar plot and (**B**) box plots of relative abundance of the most abundant gut microbiome phyla in cecum samples from standard diet (SD, red), saturated fatty acid-rich diet (SFA, blue), and monounsaturated fatty acid-rich diet (MUFA, grey) mice. (**C**) Stacked bar plot and (**D**) box plots of relative abundance of the most abundant gut microbiome phyla in colon samples from SD, SFA, and MUFA mice. (**E**) Box plots of relative abundance of the most abundant gut microbiome phyla in fecal samples from SD, SFA, and MUFA mice. Data in box plots represented with horizontal line for median, box for first and third quartiles, and whiskers for minimum and maximum values. *p<0.05, **p<0.01, Kruskal-Wallis with Dunn's multiple comparisons test, except for one-way ANOVA with Tukey's multiple comparisons test for fecal Actinobacteriota. Data from SD (n=7), SFA (n=4-5), and MUFA (n=7) cecum, colon, and fecal microbial samples.

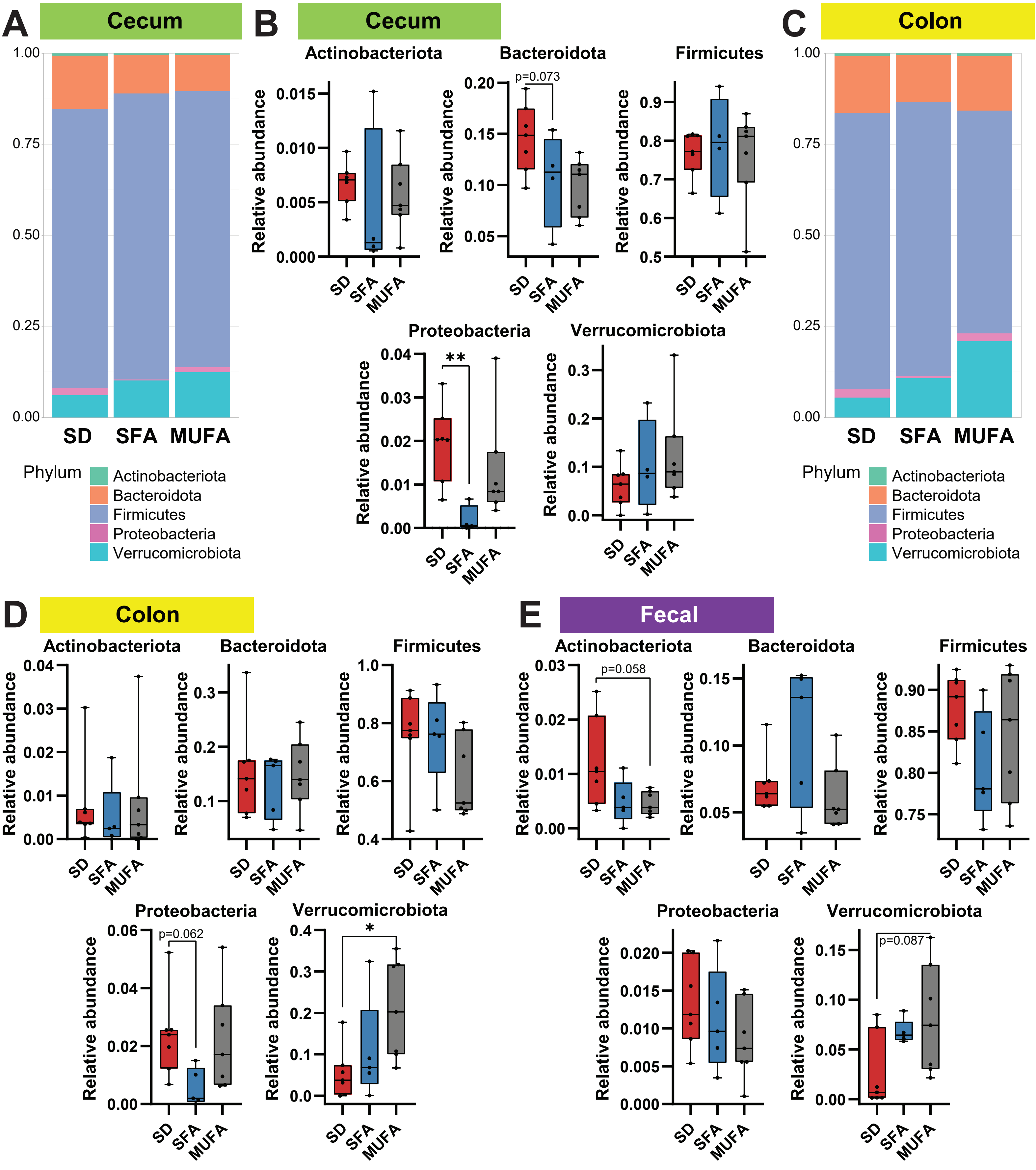

Supplementary Figure S4. Different fatty acid-rich diets associate with differential cecum and colon bacterial genera abundance. Relative abundance of the most and differentially abundant genera in (**A-D**) cecum and (**E-I**) colon microbial samples from standard diet (SD, red, n=7), saturated fatty acid-rich diet (SFA, blue, n=4-5), and monounsaturated fatty acid-rich diet (MUFA, grey, n=7) mice. Data in box plots represented with horizontal line for median, box for first and third quartiles, and whiskers for minimum and maximum values. *p<0.05, **p<0.01, ***p<0.001, Kruskal-Wallis with Dunn's multiple comparisons test.

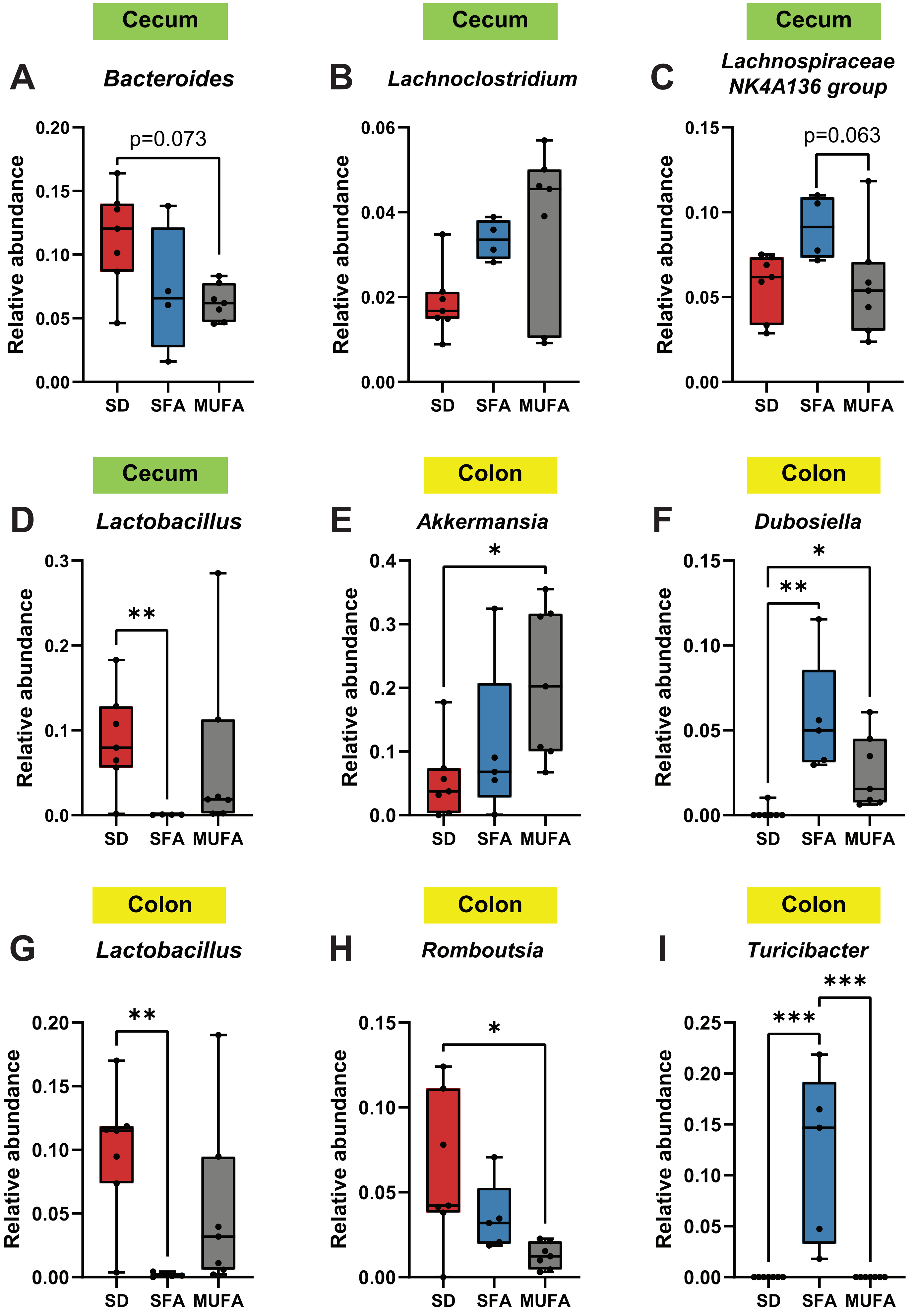

Supplementary Table S1. Differential genera abundance in by site and fatty acid-rich diets. Differential genera abundance in: **Sheet 1**: Saturated fatty acid-rich diet (SFA) versus standard diet (SD) cecum samples. **Sheet 2**: SFA versus monounsaturated fatty acid-rich diet (MUFA) cecum samples. **Sheet 3**: MUFA versus SD cecum samples. **Sheet 4**: SFA versus SD colon samples. **Sheet 5**: SFA versus MUFA colon samples. **Sheet 6**: MUFA versus SD colon samples. **Sheet 7**: SFA versus SD fecal samples. **Sheet 8**: SFA versus MUFA fecal samples. **Sheet 9**: MUFA versus SD fecal samples. lfcSE, standard error of the $\log_2$ fold-change; Padj, adjusted p-value.
